# A Bio-Inspired Drag Reduction Method of Bionic Fish Skin Mucus Structure

**DOI:** 10.3390/mi15030364

**Published:** 2024-03-06

**Authors:** Pengfei Zhao, Xin Li, Zhengjie Luo, Qihang Zhai, Ye Tian, Kaisheng Zhang, Hao Guo

**Affiliations:** 1 State Key Laboratory of Dynamic Measurement Technology, Shanxi Province Key Laboratory of Quantum Sensing and Precision Measurement, North University of China, Taiyuan 030051, China; wings215@nuc.edu.cn (P.Z.); lixinxjx@163.com (X.L.); luozzjj@163.com (Z.L.); s202106045@st.nuc.edu.cn (Q.Z.); tianye080t@163.com (Y.T.); 2 Department of Mechanical and Electrical Engineering, College of Engineering, Ocean University of China, Qingdao 266100, China; 3 Key Laboratory of Ocean Engineering of Shandong Province, Ocean University of China, Qingdao 266100, China

**Keywords:** fish skin mucus, drag reduction property, bionic design

## Abstract

Efforts to enhance the speed and reduce the energy consumption of underwater vehicles have led to the proposal of a novel mucus release structure inspired by the secretion of mucus cells on fish skin. This structure features interconnected microgrooves with excellent flexibility for adjusting to different states, effectively reducing drag through mucus release. Numerical analysis of the drag reduction performance of the mucous-releasing micro-pore structure was conducted using ANSYS Fluent 19.2 software. This structure is capable of reducing the velocity gradient near the wall and, owing to the presence of micro-pore structures, decreasing the overall compressed area, thereby achieving drag reduction effects. The experimental results revealed a drag reduction effect of 20.56% when the structure was bent at an angle of 120°. The drag reduction varied under different attitudes such as tension and compression. This mucus release structure achieves reusability through a direct mucous injection process. This research provides valuable insights for the drag reduction study of underwater vehicles, such as ships and submarines, laying a foundation for advancing the development and applications of this field in the future.

## 1. Introduction

Underwater vehicles interacting with water induce significant hydrodynamic resistance, resulting in issues such as reduced navigation speed, shortened range, and severe energy consumption [1]. Overcoming fluid pressure and friction between the fluid and the hull is imperative for underwater vehicles, where minimizing resistance is crucial. In recent years, an increasing number of researchers have conducted in-depth theoretical studies on drag reduction techniques, demonstrating widespread interest in the practical applications of this field.

Over billions of years of natural selection, living organisms have evolved effective drag reduction mechanisms to adapt to their environments, including surface microstructure patterns, microbubbles [2,3,4], hydrophobic surfaces, and surface wetting states. In the case of sharks, for instance, their low-drag riblet structure serves as an effective means to reduce drag [5]. Furthermore, the smooth surface of shark skin exhibits the capability to reduce skin friction drag and surface flow noise in turbulent boundary layers [6]. Researchers have also combined the micro-groove structure of shark skin with mucus, resulting in a remarkable drag reduction effect of up to 24.6% [7]. The integration of the microport structure can combine the drag-reducing agent with the drag-reducing microstructure, achieving a drag reduction effect superior to that of a single structure [8]. However, many drag-reducing structures have surfaces that are both tiny and intricate, leading to uneven structures during physical fabrication and resulting in a certain loss of drag reduction effectiveness. Some drag reduction methods are complex in their processes and demand high equipment requirements, thereby increasing costs.

The body surfaces of fish can release a transparent and viscous liquid through mucous cells, forming a soft material layer [9]. The surface of this soft material layer exhibits high flexibility, making it more prone to manifesting lower friction. Research indicates that the mucous is uniformly sheared and stretched in the near-wall region, resulting in a reduction in the stretching in the near-wall streaks and the strength of quasi-turbulent vortex structures in the buffer layer [10]. Experiments have also demonstrated that the mucous on shark skin, under the influence of fluid motion, can evolve into nanoscale long chains, extending into the viscous sublayer. This extension enhances the depth of the viscous sublayer, serving as a buffer layer and reducing the turbulence intensity [11]. The surface microstructure effectively slows down the rate of mucous loss. This aids in reducing friction, minimizing resistance, and protecting the organism from harmful invasions while moving through water [12]. The drag reduction effects of mucous vary among different species, and both mucous concentration and temperature have a certain impact on the drag reduction effectiveness [13]. Mucus has also been demonstrated to enhance propulsion and reduce swimming noise [14]. Similarly to the mucous drag reduction experiments, the addition of water-soluble, long-chain polymeric materials in fluid media induces a compliant effect, resulting in the formation of an elastic modulus gradient. This effectively diminishes turbulent pressure fluctuations and significantly reduces resistance [15]. The addition of drag-reducing agents such as compliant coatings, polymer drag reducers, and surfactants has been widely studied [16,17,18]. Researchers have validated the remarkable drag reduction effects by applying polymer coatings mimicking the mucous properties of fish onto the hulls of underwater vehicles. However, polymers also exhibit certain limitations, including higher costs and restricted environmental friendliness [19].

Based on the mucous secretion found on the skin of fish, this experiment introduces a mucous release structure with interconnected microgrooves designed to effectively reduce surface friction. The mucous release structure was fabricated using a template method. A simulation validated drag reduction rates at different mucous release speeds and analyzed the drag reduction mechanism. Taking advantage of PDMS’s flexible characteristics, this release structure allows for adjustments in various orientations. Experimental testing evaluated the drag reduction performance of the mucous release structure in different states, such as bending, stretching, and compression, with PDMS without microgrooves serving as a control group. This structure serves as a reference for future research on drag reduction in underwater vehicles, including ships and submarines.

## 2. Materials and Methods

### 2.1. Materials and Sample Preparation

In the experiment, hyaluronic acid was selected as the drag-reducing mucous to minimize resistance. Hyaluronic acid was procured from Shanghai McLean Biochemical Technology Co., Ltd. (Shanghai, China), with a molecular weight ranging from 800,000 to 1,500,000. Polydimethylsiloxane (PDMS) was chosen for its excellent flexibility, stretchability, high transparency, corrosion resistance, ease of preparation, and controllable shape. PDMS and the curing agent were obtained from Dow Corning, Midland, MI, USA. For mucous preparation, 0.1 g of hyaluronic acid was accurately weighed and added to 100 mL of distilled water. Mixing was achieved with a chemically inert cylindrical glass stirring rod, resulting in the eventual formation of a clear and viscous mucous solution. The stirring speed was set at 200 revolutions per minute, with a duration of 10 min. In the experiment, a syringe was employed to draw the mucous, which was then injected into the mucous release structure.

**Figure 1:**
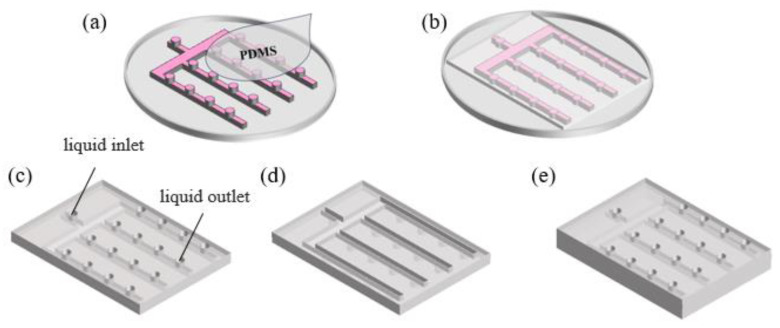
Schematic representation of the mucous release structure fabrication process. (**a**) Template and PDMS pouring demonstration. (**b**) Conceptual illustration after PDMS casting. (**c**) Frontal display of microgroove-interconnected structure. (**d**) Dorsal view of microgroove-interconnected structure. (**e**) Diagram of the mucous release structure.

In the experimental setup, a mucous release structure with internally interconnected microgrooves was designed, and the fabrication process is illustrated in Figure 1, employing the commonly utilized template method. Figure 1a displays the template structure, crafted through etching techniques. PDMS and the curing agent were mixed in a 10:1 ratio and thoroughly degassed in a vacuum chamber to eliminate bubbles, yielding a transparent mixture. This mixture was cast onto the template, as depicted in Figure 1b, and cured for two hours at 80 °C. Following the solidification of the composite, the flexible PDMS was peeled off the template, with its front side revealed in Figure 1c and the reverse side in Figure 1d. Notably, the backside exhibited interconnected microgroove structures, while the front side presented an array of densely distributed small orifices. The isolated orifice at the convergence of the microgrooves served as the inlet for fluid injection, simulating mucous secretion in fish skin. The remaining orifices, arranged in an array, functioned as outlets interconnected with the microgrooves, mimicking the mucous-secreting pores on fish skin. Additionally, a smooth-surfaced PDMS was affixed to the backside of the PDMS with microgrooves and micropores, as illustrated in Figure 1e. The adhesion between the two PDMS layers, facilitated by their inherent affinity, completed the fabrication of the structure. The structure incorporated interconnected microgrooves that converged at an inlet, where mucous was injected, filling the entire microgroove structure. The mucous then flowed out through an outlet located above the neatly arranged microgrooves, covering the surface of the structure.

### 2.2. Drag Reduction Experiment

**Figure 2:**
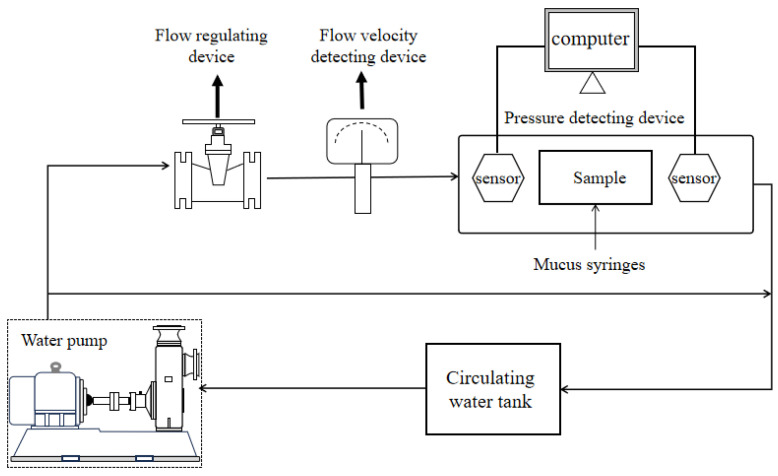
Drag reduction performance test device.

The drag reduction performance of the materials was experimentally evaluated using the drag reduction test setup illustrated in Figure 2. The circulating water medium was maintained at 20 °C, and it entered the pressure detection device through a water pump, with water velocity controlled by a flow regulator. Within the pressure detection device, two sensors were employed to collect pressure values at both ends of the sample [20]. The flow rate of mucous within the sample was controlled using a mucous injection device. Subsequently, a data processing unit was employed for comprehensive analysis and calculations to determine the pressure drop across the sample channel. Multiple sets of experimental results were compared, and drag reduction rates under different flow rates and conditions were computed using Equation (1).

(1) $$DR=\frac{F_{1}-F_{2}}{F_{1}}\times 100\%$$

Here, *F*_1_ represents the surface resistance on the smooth, uniform PDMS surface without the mucin release structure, and *F*_2_ represents the surface resistance on the mucin-releasing structure. All data were measured after achieving a stable water velocity, and each data point underwent multiple repeated measurements.

### 2.3. Numerical Simulation

**Figure 3:**
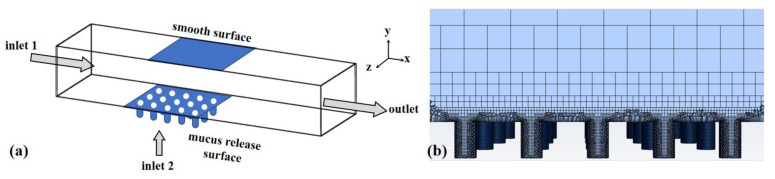
(**a**) The computational domain model. (**b**) The grid division diagram.

To validate the drag reduction performance of the mucous release structure, a rigid material, aluminum, was used to establish the model. The simulation did not consider the influence of flexibility on the drag reduction performance. To obtain more accurate drag reduction results, the smooth surface and the mucous release surface were compared under the same computational area and identical flow velocity. A simulation model was constructed in ANSYS Fluent 19.2, and the calculated boundary conditions are shown in Figure 3a. The dimensions of the calculation domain model were length (X) × width (Y) × height (Z): 500 mm × 50 mm × 82 mm. To avoid the influence of the entrance and exit, the entrance distance: the length of the nonsmooth zone: the exit distance was set at 4:1:5. In it, the microhole size and spacing were the same as the physical dimensions. The inlet was the velocity inlet, the outlet was the pressure outlet, and the two sides of the wall were symmetrical. The fluid medium was liquid water with a density of 1.0 × 10^3^ kg·m^−3^ and a dynamic viscosity coefficient of 1.0 × 10^−3^ Pa·s. The dimensionless Reynolds number represents the ratio of inertial forces to viscous forces. The formula for calculating the Reynolds number (Re) is given by Re = ρuL/µ, where ρ is the fluid density, u is the fluid average velocity, L is the characteristic length, and µ is the viscosity coefficient. In this simulation, the Reynolds number was 124,000. A pressure-based solver was employed to solve the equations, with the turbulence model set to RNG (Re-Normalisation Group) k-epsilon. The “enhanced wall treatment” feature was utilized to handle the near-wall region. The SIMPLE algorithm was used for numerical simulation. The discretization scheme adopted a second-order upwind scheme. By utilizing the flow field analysis preprocessing software ICEM 19.2 for model grid generation, we employed grid quantities of 250,000, 320,000, and 430,000 to verify the grid independence of the flow field calculations. As shown in Table 1, we selected the drag coefficient and Strouhal number as the indicators to evaluate grid convergence, with the differences calculated using the results obtained with 430,000 grid cells as a reference. When the grid quantity was 320,000, the drag coefficient error was 0.2462%, and the Strouhal number error was 0.04%. This indicates that, at this grid quantity, the results of the flow field had stabilized. Thus, choosing 320,000 grid cells ensures both the accuracy of the results and the computational time efficiency. Figure 3b illustrates the grid partitioning with a quantity of 320,000 cells. On the mucus release surface, the grid was smaller, allowing for more precise calculations.

For ease of calculation and without considering fluid–structure coupling, fluid dynamics (CFD) simulation was selected. Water flowed in from inlet 1 and exited from the outlet, while mucous entered the model through inlet 2 below, spreading on the mucous release surface. The size and spacing of the circular holes on the mucous release surface were consistent with the physical model. In the simulation, the water flow velocity at inlet 1 was set to 2 m/s, and the mucous flow velocity at inlet 2 was varied to investigate the relationship between the mucous velocity and drag reduction rate. In the simulation, the steady-state calculation was set with 4000 iterations, while the transient calculation employed a time step of 1 × 10^−4^ s, with 10 iterations per time step. The drag reduction rate of the mucous release surface compared to the smooth surface could be calculated using Equation (1). In the simulation results, the total resistance (F) was composed of pressure difference resistance (F_dp_) and viscous resistance (F_v_) [20]. The main factors affecting pressure difference resistance include cross-sectional area, object shape, and the orientation in the flow field. The primary factors influencing viscous resistance are surface roughness, relative velocity, and friction coefficient [11].

## 3. Results and Analysis

### 3.1. Mucus Release Structure

**Figure 4:**
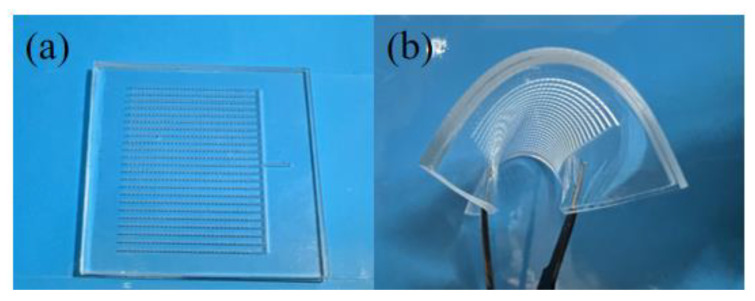
(**a**) Physical display. (**b**) Flexible display.

The physical representation is shown in Figure 4, with Figure 4b illustrating the flexibility. In the diagram, the groove width is 0.2 cm, the spacing between adjacent grooves is 1.8 cm, and the diameters of the inlet and outlet ports are both 0.3 cm. The distance between adjacent outlet ports above the same groove is 0.92 cm. The groove depth and outlet port depth are both 0.45 cm, and the thickness of both layers of PDMS is 0.9 cm. Mucous was injected into the microgrooves, and when the mucous release structure was in different states, such as stretching, compression, and bending, mucous was released from the micropores, altering the magnitude of resistance. The amount of mucous released varied under different orientations of the mucous release structure, affecting the resistance. The mucous release structure aimed to form a smooth membrane on the surface of an object, reducing frictional resistance when water is flowing over it. Mucus can absorb and disperse energy in turbulent flow, smoothing fluid motion [19]. This helps to diminish turbulence caused by irregular fluid motion, thereby reducing turbulent resistance.

### 3.2. Simulated Analysis

**Figure 5:**
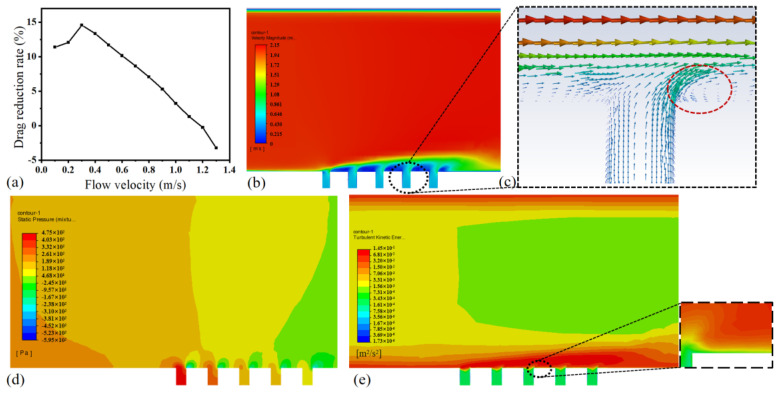
(**a**) Graph depicting the relationship between mucous flow velocity and drag reduction rate. (**b**) Velocity cloud diagram of the x-y cross-section, where the upper section represents the smooth surface and the lower section represents the mucous-releasing surface. (**c**) Velocity vector distribution in the near-wall area. (**d**) Static pressure cloud diagram of the x-y cross-section. (**e**) Turbulent kinetic energy map of the x-y cross-section.

To minimize excessive mucous loss and enhance drag reduction rates, simulation analysis was employed to identify the optimal mucous release velocity. In the simulation, a constant water flow velocity of 2 m/s was prescribed, while the mucous flow velocity at the inlet 2 was systematically varied. With the increase in mucous flow velocity, the drag reduction rate initially rose and subsequently decreased, as illustrated in Figure 5a. When the mucous velocity is significantly lower than the water flow velocity, it may not effectively adhere to the structure’s surface, leading to potential loss and suboptimal drag reduction effects. Within a specific velocity range, the mucous is more prone to forming a lubricating layer, reducing frictional resistance. However, excessively high mucous velocity can introduce a certain level of resistance, resulting in a decline in drag reduction effectiveness [10].

Figure 5b displays the velocity distribution map on the x-y plane, with the smooth surface above and the mucous-releasing surface below. It can be observed from the graph that, at the same distance in the y-direction, the velocity growth on the biomimetic surface was relatively gradual, with a smaller velocity gradient and viscous resistance [21]. Therefore, the mucous release structure surface contributes to an increase in boundary layer thickness from the solid surface to the predominant flow velocity, thus effectively reducing resistance. As indicated by the red dashed box in Figure 5c, low-speed vortices were generated near the wall surface, decelerating the local fluid velocity, thereby reducing frictional resistance and achieving an overall drag reduction effect. The improvement in lubrication was mainly due to liquid–liquid repulsion, making the liquid–solid contact line move more smoothly in the horizontal direction (x-direction), resulting in a reduction in viscous resistance [22].

The static pressure contour map shown in Figure 5d reveals that the prominent micro-hole structure in this configuration hinders the movement of the flow field, resulting in significant wall pressure differences and altering the dynamic state of the flow field, thereby generating small-scale vortices. However, the existence of the micro-hole structure simultaneously reduced the frictional resistance between the flow field and the sample surface, attributed to the reduction in contact area [23]. The turbulence intensity of the mucous-release surface structure was observed to be lower than that of the smooth surface, as evidenced by the turbulent kinetic energy plot in Figure 5e. This is conducive to reducing velocity fluctuations. The velocity cloud diagram in Figure 5b also demonstrates that the low-speed region of the mucous-release surface structure exceeded that of the smooth surface. This resulted in a slower exchange of momentum and kinetic energy during fluid motion, thereby reducing surface drag and achieving drag reduction. The thickness of the high-turbulence region on the microstructured surface was greater than that on the smooth surface. The turbulence intensity decreased with the distance from the wall, and the turbulence intensity far from the wall was hardly affected by the surface microstructure [24].

### 3.3. Experimental Analysis of Drag Reduction

**Figure 6:**
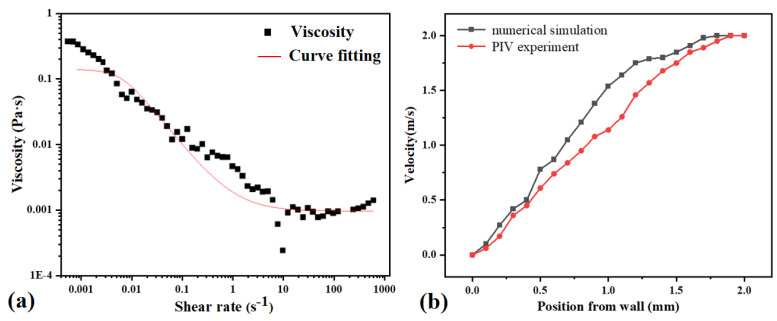
(**a**) Rheological property testing results of mucus. The solid line represents the prediction of the Carreau model. (**b**) The distribution curve of the dimensionless average velocity along the normal direction.

During the experimental procedure, rheological performance testing of the mucus was conducted using a rheometer (AR-G2, TA Instruments, New Castle, DE, USA). Examining the results in Figure 6a, the steady-state curve exhibits characteristics consistent with the Carreau model: the viscosity of the mucus showed little variation at low shear rates, resembling that of a Newtonian fluid; as the shear rate increased, the mucus viscosity decreased significantly, resembling that of a non-Newtonian fluid; and when the shear rate reached a certain value, the magnitude of viscosity variation decreased, resembling that of a Newtonian fluid. It can be observed that at higher shear rates, the viscosity was lower, exhibiting shear-thinning characteristics [25]. This can be attributed to the fact that, at elevated shear rates, molecules have sufficient energy to rearrange, resulting in a relatively more fluidic behavior of the fluid. The shear-thinning characteristics facilitate the easier flow of mucus while concurrently reducing friction, thereby imparting a favorable lubricating effect.

In the experiment, we dispersed 10 μm tracer particles from the Dantec brand in the flow field and recorded, as well as analyzed, the velocity distribution using particle image velocimetry (PIV). PIV experiments encompass hardware components such as lasers, high-speed cameras, and a circulation system for inducing motion in the fluid medium. Additionally, imaging software and velocimetry software systems were employed. Following the completion of the experiment, particle images were imported into the PIV processing software, MicroVec 3.6.1 (Beijing China), where the built-in cross-correlation algorithm was utilized for image analysis, thereby extracting the velocity vector field of the flow field. Figure 6b illustrates the velocity distribution curve along the normal direction to the surface of the mucous release structure in the PIV experiment. In this experiment, the water flow velocity was set at 2 m/s, and the mucous flow velocity was 0.3 m/s. The experimental results indicate a relatively slow velocity variation with a smaller velocity gradient compared to the simulation, suggesting a greater thickness of the boundary layer.

**Figure 7:**
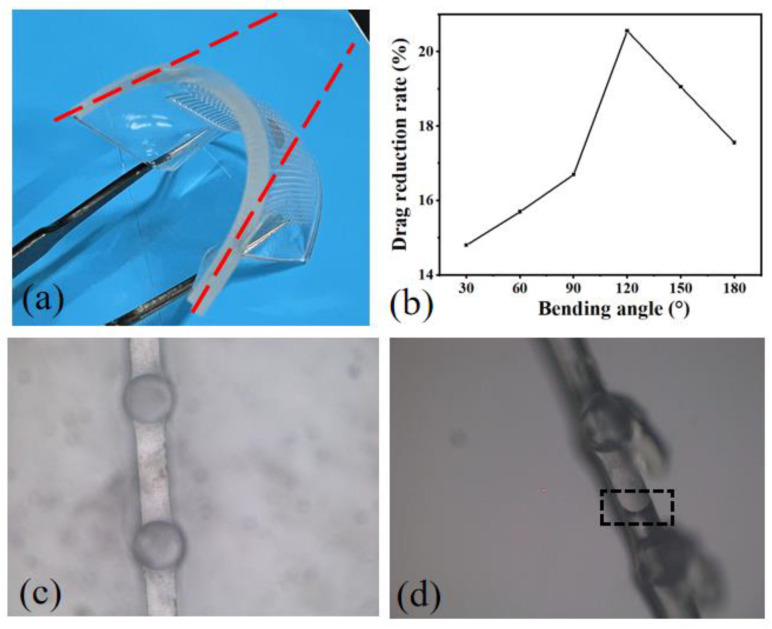
Impact of bending angle on drag reduction. (**a**) Photographic representation of the structure under bending conditions. (**b**) Drag reduction rates under different bending angles. (**c**) Microscopic observation before bending. (**d**) Microscopic observation after bending.

To verify the drag reduction performance of this structure under different conditions for subsequent application in various scenarios, experiments were conducted to test the drag reduction rate under different bending angles, stretching degrees, compression degrees, and different water flow angles. The following experimental results represent the average of multiple repeated tests, with a deviation within 5% for each trial. This approach of repetitive testing ensures the reliability and stability of the experimental data. The water flow velocity was fixed at 2 m/s, and the mucous release velocity was set at 0.3 m/s, the value that yielded the best drag reduction in the simulation. The bending angle was defined as the angle between the extensions of the two red dashed lines, with 180° representing no bending. The experiments tested drag reduction rates for bending angles ranging from 30° to 180°, as shown in Figure 7b. The drag reduction rate increased with the increasing bending angle, reaching a maximum of 20.56% at 120°, indicating optimal drag reduction effects. Microscopic observations of the microchannels and outlet conditions were made before and after bending, as shown in Figure 7d. It can be observed that there were bubbles in the microchannels, and the black dashed box encircled the gas–liquid interface. Excess mucous flowed out from the outlet, indicating a higher amount of mucous on the surface of the structure under bending conditions. The drag reduction rate without bending was approximately 17.56%, and compared to the simulation results, the physical model made with flexible PDMS demonstrated better drag reduction effects. Flexible materials can slow down velocity changes, reduce turbulence generation, and achieve drag reduction effects [26].

**Figure 8:**
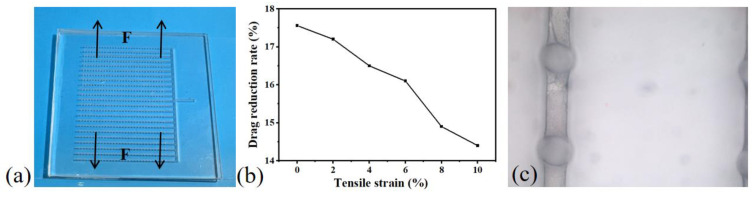
Impact of stretching degree on drag reduction. (**a**) Representation of the stretching direction. (**b**) Drag reduction rates under different stretching degrees. (**c**) Microscopic observation after stretching.

In the experiment, the structure was subjected to stretching in the direction perpendicular to the grooves. Figure 8c shows a microscopic schematic during stretching, revealing the transformation of the liquid surface from spherical to ellipsoidal. The stretching degree is defined as the ratio of the stretching length to the original length. Since excessive stretching can lead to structural breakage from the grooves, the experiment tested the drag reduction effect within a ten percent stretching degree. As shown in Figure 8b, the drag reduction rate decreased with the increasing stretching degree. This is attributed to the reduced relative coverage area of mucous, and the slight squeezing of the openings during stretching caused shape changes.

**Figure 9:**
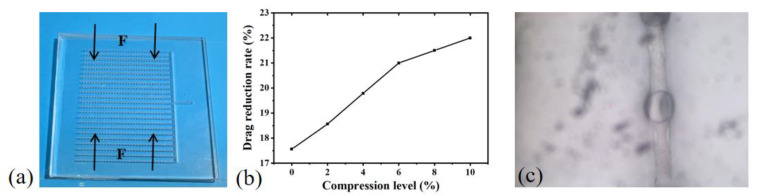
Impact of compression degree on drag reduction. (**a**) Representation of the compression direction. (**b**) Drag reduction rates under different compression degrees. (**c**) Microscopic observation after compression.

Compression was applied to the structure in the direction perpendicular to the grooves, as shown in Figure 9a. Figure 9c shows a microscopic schematic during compression, indicating the transformation of the liquid surface from spherical to ellipsoidal. The compression degree is defined as the ratio of the compressed length to the original length. Figure 9b presents drag reduction rates within a 10% compression degree, showing an increase in the drag reduction rate with the growing compression degree. This is attributed to the reduction in groove space and the extrusion of mucous as the compression degree increased, favoring a decrease in drag.

**Figure 10:**
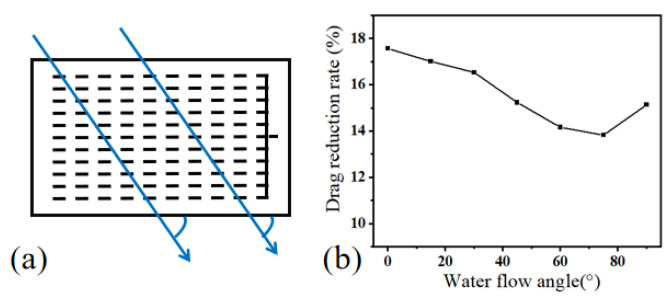
Impact of water flow angle on drag reduction performance. (**a**) Schematic diagram of the angle between water flow direction and structure. (**b**) Drag reduction rate at different water flow angles.

In this experiment, the water flow angle is defined as the angle between the direction of water flow and the grooves, as illustrated in Figure 10a. In the experiment, the mucous release structure exhibited a certain degree of curvature, ensuring the discharge of mucus from the pores. With the curvature angle held constant at 120°, the impact of the water flow angle on the drag reduction performance was investigated. As depicted in Figure 10b, within the range of 0–90° for the water flow angle, the drag reduction rate initially decreased and then increases with the augmentation of the water flow angle. This outcome implies that the optimal drag reduction effect was achieved when the water flow velocity was parallel to the mucous grooves.

## 4. Conclusions

In order to reduce the hydrodynamic resistance of underwater vehicles, inspired by the mucous secretion on the skin of fish, a biomimetic mucous-release drag reduction surface was investigated. Drawing conclusions from both experimental and numerical simulations, the following findings emerged:

(1) A simulation analysis of mucous release on the surface of the structure was conducted using ANSYS Fluent 19.2. Under the conditions of a water flow velocity of 2 m/s and a mucous velocity of 0.3 m/s, the maximum drag reduction speed was observed to be approximately 15%. It is noteworthy that, with a fixed water flow velocity, the drag reduction effect exhibited an increasing trend, followed by a decrease as the mucous velocity increased.
(2) This mucous release structure can control near-wall flow, reduce near-wall velocity gradients, and increase boundary layer thickness.
(3) Flexible physical models of the mucous release structure were fabricated using PDMS, and the drag reduction performance was tested under different conditions, including bending angles, stretching levels, compression levels, and various water flow angles.
(4) The drag reduction effect varied under different bending angles, with the optimal reduction achieved at a bending angle of 120°, reaching 20.56%. When the stretching level was within 10%, the mucous outflow gradually decreased, leading to a reduced drag reduction effect. Within a compression level of 10%, an increased compression level resulted in more mucous extrusion, leading to a better drag reduction effect. The drag reduction effect initially decreased and then increased with an increase in the water flow angle.

This structure relies on the injection of mucus under pressure to release mucus, and the drag reduction process mainly consumes mucus, while the release structure is reusable. Compared to traditional drag reduction techniques with higher energy consumption, this method has significant advantages in terms of energy consumption. Firstly, the energy required for mucus injection is relatively low, and energy consumption can be minimized by optimizing the injection system and controlling the frequency of mucus release. Secondly, the reusability of the release structure minimizes energy consumption, thereby reducing energy costs and environmental impact. This structure demonstrates the potential to provide effective drag reduction for underwater vehicles, significantly enhancing their economic efficiency and presenting extensive application prospects.

## Figures and Tables

**Table 1:** The comparison of computational results under different grids.

Number of Grids (Ten Thousand)	Drag Coefficient Error	Strouhal Number Error
25	1.4281%	0.8%
32	0.2462%	0.04%
43	--	--

## Data Availability

The data supporting the reported results by the authors can be sent by e-mail. The data are not publicly available due to confidentiality request.
